# Adipokine Leptin Co-operates With Mechanosensitive Ca^2 +^-Channels and Triggers Actomyosin-Mediated Motility of Breast Epithelial Cells

**DOI:** 10.3389/fcell.2020.607038

**Published:** 2021-01-06

**Authors:** Anna Acheva, Tytti Kärki, Niccole Schaible, Ramaswamy Krishnan, Sari Tojkander

**Affiliations:** ^1^Section of Pathology, Department of Veterinary Biosciences, University of Helsinki, Helsinki, Finland; ^2^Department of Applied Physics, School of Science, Aalto University, Espoo, Finland; ^3^Beth Deaconess Medical Center, Harvard Medical School, Boston, MA, United States

**Keywords:** adipokines, actomyosin, calcium, actin, epithelium

## Abstract

In postmenopausal women, a major risk factor for the development of breast cancer is obesity. In particular, the adipose tissue-derived adipokine leptin has been strongly linked to tumor cell proliferation, migration, and metastasis, but the underlying mechanisms remain unclear. Here we show that treatment of normal mammary epithelial cells with leptin induces EMT-like features characterized by higher cellular migration speeds, loss of structural ordering of 3D-mammo spheres, and enhancement of epithelial traction forces. Mechanistically, leptin triggers the phosphorylation of myosin light chain kinase-2 (MLC-2) through the interdependent activity of leptin receptor and Ca^2+^ channels. These data provide evidence that leptin-activated leptin receptors, in co-operation with mechanosensitive Ca^2+^ channels, play a role in the development of breast carcinomas through the regulation of actomyosin dynamics.

## Introduction

Breast cancer is the most common malignant disease among the female population and has the highest mortality rate, in most cases due to secondary tumors (Bray et al., 2018). Lately, a growing body of evidence has shown that the tumor microenvironment has an important role for the development of breast tumors (Arendt and Kuperwasser, 2015; Seo et al., 2015). Particularly the role of the adipose tissue, which is a main component of the breast stroma, has been pointed out to be leading cause for oncogenesis and cancer progression (Wang et al., 2012; Picon-Ruiz et al., 2016, 2017). However, the specific mechanisms of how the adipose tissue drives the initial stages of cancer development remain elusive.

Adipose is not only a passive storage of lipids, but has also dynamic secretory and metabolic functions, which are dysregulated in obese individuals and during cancer progression (Vona-Davis and Rose, 2007; van Kruijsdijk et al., 2009; Schweizer et al., 2015). At the same time, a striking correlation between obesity and the invasiveness of the breast cancers in postmenopausal women has been described (Neuhouser et al., 2015; Osman and Hennessy, 2015). For obese women, the risk to die from the breast cancer is more than 2.5 times higher than in lean patients (Neuhouser et al., 2015). As a contributor to breast cancer progression, the adipose tissue has been mainly thought to create local inflammatory reactions or to act as a source of energy for the cancer cells via fatty acid release (Pan et al., 2018). Besides that, adipose endocrine factors such as the adipokines leptin and adiponectin are known to correlate with the invasive phenotype and metastasis of breast cancer (Vona-Davis and Rose, 2007; Libby et al., 2014; Pan et al., 2018; Andò et al., 2019). Leptin is a product of the obese (*ob*) gene and traditionally regulates energy metabolism and satiety through stimulation of the central nervous system. In breast cancer, it has pro-mitogenic functions as it induces PI3K/Akt survival pathway and up-regulates genes associated with metastasis (Yan et al., 2012; Kato et al., 2015). Leptin also increases the proliferation of normal breast epithelial cells (Hu et al., 2011; Tenvooren et al., 2019). In contrast, adiponectin is an anti-proliferative and negatively correlated with cancer invasion and metastasis (Jardé et al., 2011). Adiponectin has also effect on the metabolism of reactive oxygen species, inhibits cell proliferation and reduces tumor angiogenesis (van Kruijsdijk et al., 2009; VanSaun, 2013). However, in the obese individuals adiponectin levels are low and that in combination with high leptin levels correlates with larger tumors and higher tumor grade (Vona-Davis and Rose, 2007). Additionally, adipokines have been suggested to induce changes in the cell morphology and trigger epithelial-to-mesenchymal transition (EMT), a process closely related to cancer progression (Yan et al., 2012; Kato et al., 2015). Based on the previous observations, there is clearly a strong need to investigate in more detail the mechanisms, which adipocyte cells utilize to initiate tumorigenesis and invasion.

Initiation of cancer metastasis starts with the onset of invasion. During invasion tumor cells detach from the primary tumor site due to weakened cell-cell adhesions and alter the extracellular matrix for efficient migration (Scully et al., 2012). Under normal conditions, epithelial cell sheets are kept intact by actomyosin bundles, which connect to the extracellular matrix (ECM) and to the cadherin-based cell-cell contacts for the maintenance of tensional balance (Maruthamuthu et al., 2011; Wu et al., 2014; Lecuit and Yap, 2015). Prior to invasion, these actomyosin forces are unbalanced, leading to loss of epithelial integrity and abnormal cell-substrate forces that cancer cells may utilize for migration and to modify their environment more permissive for the invading cells (Mierke et al., 2008, 2011; Kraning-Rush et al., 2012; Aung et al., 2014). Actomyosin contractility is driven by the myosin II motor protein and is usually triggered by the phosphorylation of myosin light chain-2 (MLC-2) (Somlyo and Somlyo, 2000). The dynamics of contractile actomyosin bundles is regulated by several mechanosensitive pathways, including Rho/ROCK pathway, which is probably the most well understood and also targeted by stromal alterations (Bershadsky et al., 2003; Huang et al., 2004). Many of the actomyosin-regulating kinases, such as myosin light chain kinase, MLCK, and Ca^2+^ -dependent kinases, CaM kinases, are also reliant on calcium and therefore local Ca^2+^ influxes through specific channels can play a major role in the activity of actomyosin structures both in single migrating cells and in epithelial sheets (Somlyo and Somlyo, 2000; Clark et al., 2006, 2007; Miranda et al., 2010; Tojkander et al., 2015, 2018; Haws et al., 2016; Rajakylä et al., 2020). In addition, Ca^2+^ signaling is known to regulate migration of normal and breast cancer cells (Wei et al., 2009; Iamshanova et al., 2017) and certain mechanosensitive Ca^2+^ channels, particularly the Transient Receptor Potential (TRP) and Orai1 families, are overexpressed in breast cancer and associated with poor clinical prognosis (Bolanz et al., 2008; Dhennin-Duthille et al., 2011). As Ca^2+^ signaling is directly involved in the induction of EMT (Dowd et al., 2017), it may trigger dissemination and invasion of cancer cells through cytoskeletal changes. However, it is not yet known whether the adipose-secreted factors and Ca^2+^ signaling interact to facilitate epithelial cell scattering prior to invasive stage.

In the current work, we show that adipose signaling, and in particularly leptin signaling, can alter morphology and migration capability of normal mammary epithelial cells. These features were associated with induction of EMT-related proteins and loss of normal 3D mammosphere structures. Additionally, treatment of mammary epithelial cells with adipokines triggered MLC-2 phosphorylation, subsequently leading to higher actomyosin forces that may play a role in cell motility. Myosin 2-mediated contraction, induced by leptin, at least partially took place in an AMPK-dependent manner and required the interplay in between leptin signaling and calcium influx. These data suggest that leptin is involved in the regulation of actomyosin dynamics by switching Ca^2+^ influx of the cells. Our findings thus support the previous observations on the association of excessive leptin in breast cancer progression and reveal a novel, co-operative action of the leptin receptor and mechanosensitive Ca^2+^ channels, which could favor the development of invasive breast cancer.

## Results

### Leptin Triggers Motility and EMT-Like Phenotype in Normal Breast Epithelial Cells

**FIGURE 1:**
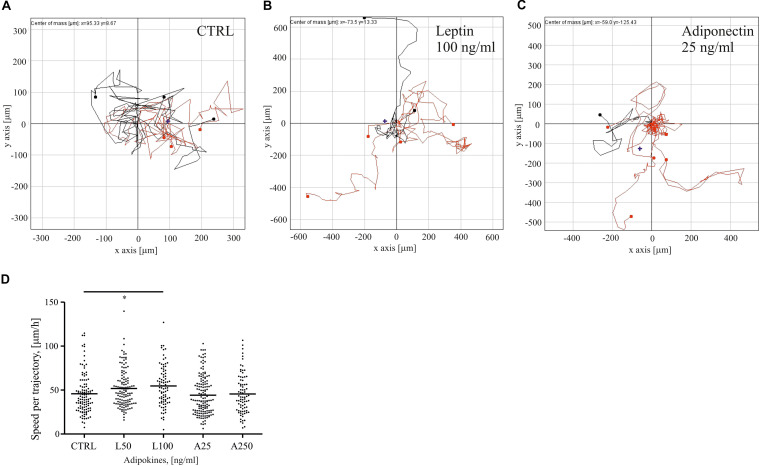
Leptin triggers breast epithelial cell migration. MCF10A cells were followed for their migratory features after 24 h of treatments. Representative traction plots of **(A)** control in serum-free medium; **(B)** Leptin 100 ng/ml; and **(C)** Adiponectin 25 ng/ml; **(D)** median values of cell speed per trajectory calculated with Cell IQ Analyser (CM technologies, Tampere, Finland), results are from at least 71 cells per condition. *n* = 2, *p < 0.05 Kruskal-Wallis test.

Adipose tissue-secreted cytokines have been reported to affect motility and proliferation of breast epithelial cells (Treeck et al., 2008; Jardé et al., 2011). To understand how two distinct adipokines, i.e., leptin and adiponectin, could impact motility of normal mammary epithelial cells, we treated sparsely growing MCF10A cells with these distinct adipokines and tracked their migration in time. Single cell cultures, exposed to serum starvation, were left untreated or treated with either 50–100 ng/ml of leptin or 25–250 ng/ml of adiponectin, and were imaged over 24 h. The trajectories of migratory cells were tracked with the Cell IQ Analyser program (Figures 1A–C). MCF10A cells, treated with higher dose of leptin, demonstrated statistically significant increase in cell motility (Figures 1B,D). In contrast, adiponectin-treated cells did not show significant difference in the migration speed in comparison to ctrl sample (Figures 1C,D).

**FIGURE 2:**
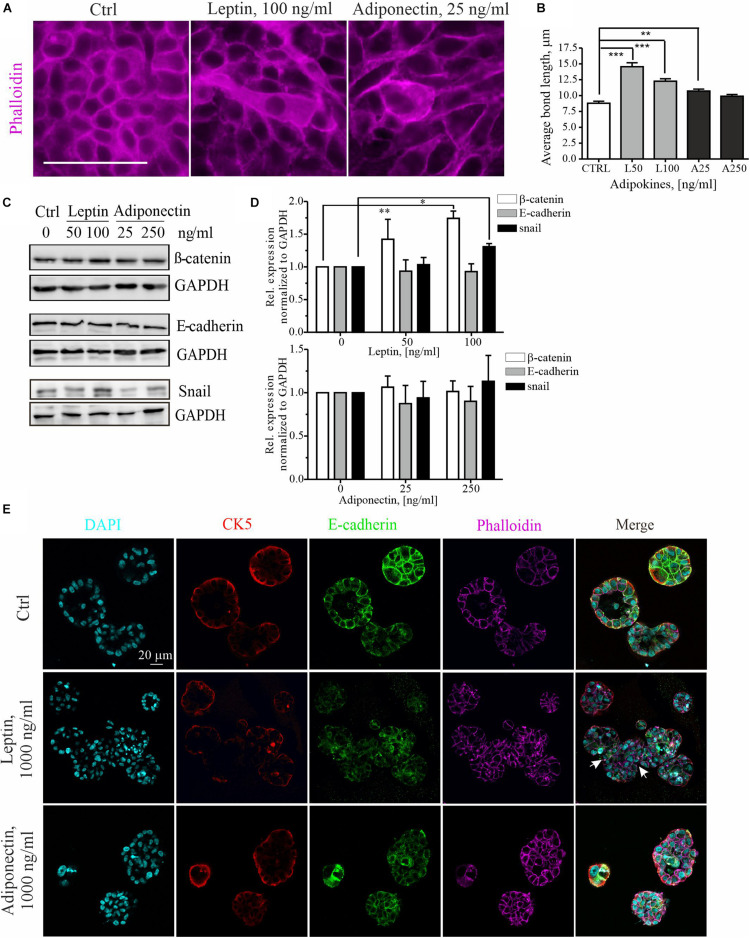
Leptin treatment disrupts the epithelium and induces EMT markers. **(A)** Ctrl, leptin- and adiponectin-treated MCF10A cell monolayers after 72 h in culture. Treatment with 100 ng/ml of leptin disrupted epithelial sheet morphology and led to elongation of the cells within the monolayer. Actin cytoskeleton was visualized with phalloidin. Scale bar 50 μm. **(B)** Monolayer morphology was evaluated from the phalloidin stainings by using the Tissue analyzer plugin for Image J. Average length of the cell-cell bonds after leptin and adiponectin treatments was assessed. **p < 0.01, ***p < 0.001, One-way ANOVA, Tukey’s post-test. **(C)** Cellular lysates of ctrl, leptin- and adiponectin-treated cells were analyzed by Western blotting. Specific antibodies against β-catenin, E-cadherin and snail were utilized. GAPDH acts as a loading control. **(D)** Western blot quantification related to Figure 2C. *n* = 3 (for snail) and *n* = 4 (for E-cadherin and β-catenin), Student’s *t*-test, *p < 0.05, **p < 0.01). **(E)** MCF10A cells were culture in 3D matrigel for 14 days, after which they were fixed and used for immunofluorescence stainings. Specific antibodies against CK5 and E-cadherin were utilized. Phalloidin was used to detect actin cytoskeleton and DAPI nuclei. CK5 stains the outer, myoepithelial cell layer. Images show the morphology of 3D spheroids from MCF 10A cells treated with leptin or adiponectin (1,000 ng/ml for both). Leptin was changing the spheroid structure to irregular (indicated with white arrows). Scale bar 20 μm.

To further examine the cytoskeletal changes associated with leptin-triggered migratory cells, we assessed the morphology of these cells within mammary epithelial sheets. Treatment of MCF10A cell cultures with higher dose of leptin, induced clear morphological changes in the epithelial cells (Figure 2A). The elongated cell shape in these epithelial sheets was analyzed by measuring the average length of cell-to-cell bonds within the monolayers with Image J plugin Tissue Analyzer as described in section “Materials and Methods” and (Aigouy et al., 2016). Based on this analysis, leptin-treated cells were significantly elongated in comparison to the control and adiponectin-treated cells (Figure 2B).

Concomitant with the morphological changes, an increase in the mesenchymal markers β-catenin, snail, N-cadherin and slug was detected upon leptin treatment, while E-cadherin levels remained constant (Figures 2C,D and Supplementary Figures S1A,B, S2A). Leptin treatment was associated with occasional internalization of the epithelial marker E-cadherin (Supplementary Figure S2A). Adiponectin treatment, however, had not significant effect on the expression of these EMT-linked markers.

Finally, we studied the impact of adipokine treatments on the 3D morphology of the mammary spheroids. For this, MCF10A cells were cultured within Matrigel that contains extracellular matrix components and resemble the physiological conditions. Long-term cultures of the 3D spheroids also allowed better screening of the later effects of adipokine signaling on the mammary epithelial cells. As shown in the upper panel of Figure 2E, control MCF10A cells in 3D Matrigel form polarized structures with hollow lumens, similar to the mammary acini (LaBarge et al., 2009, 2013). However, 3D spheroid cultures, in the presence of leptin, displayed larger and irregular structures (Figure 2E, middle panel, white arrows indicate the irregular structures; additional images from the leptin-treated spheroids in Supplementary Figure S1E show shape variation). Treatment of spheroids in 3D with leptin was in 10 times higher range to produce an effect (Jardé et al., 2011). Analyses of the volume of these spheroids showed a slight increase in the median size of the spheroids upon 1,000 ng/ml of leptin (Supplementary Figure S1C). Interestingly, the adiponectin treatment had a suppressing effect on the mammosphere growth and these spheroids were less spherical (Supplementary Figures S1C,D).

In summary, leptin induces morphological changes in normal breast epithelial cells to induce their migratory features possibly through partial EMT, while adiponectin had no significant impact on these cellular properties. In line with the previous observations, excessive leptin may thus favor cancer cell migration.

### Leptin Induces Actomyosin-Mediated Cellular Forces

**FIGURE 3:**
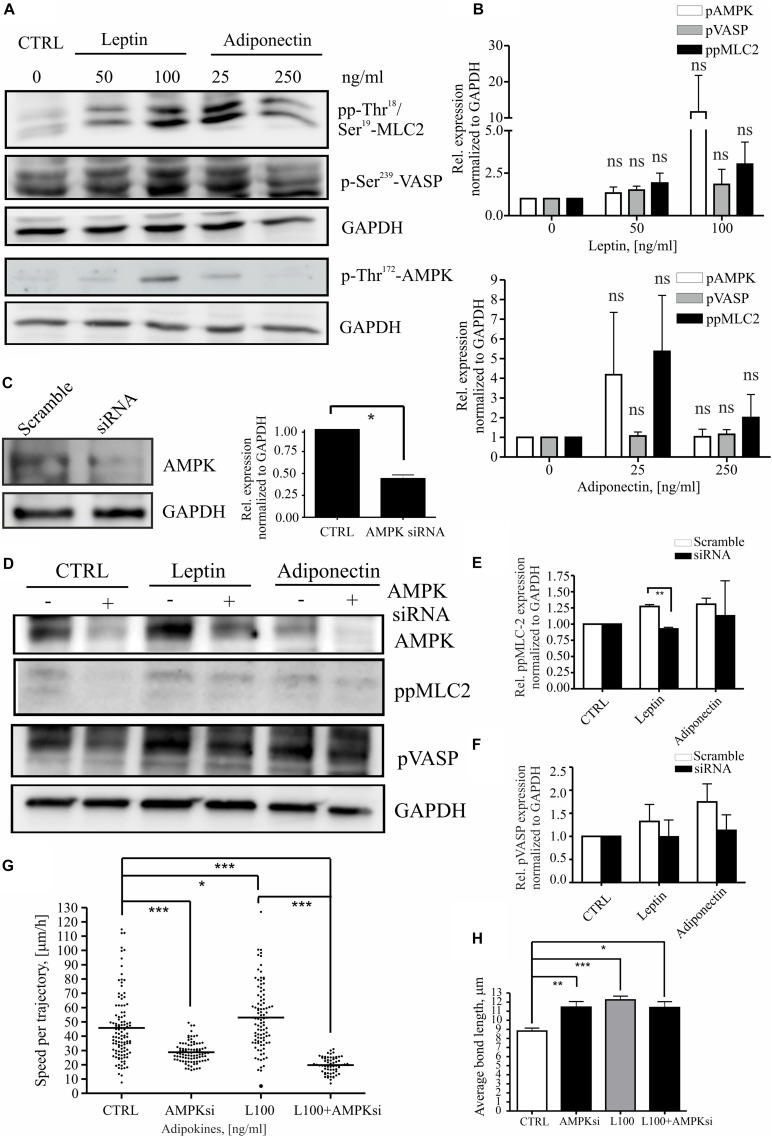
Leptin induces MLC-2 phosphorylation. **(A)** Western blot analysis of cellular lysates from MCF10A cells. Specific antibodies against pp-Thr18/Ser19-MLC2, p-Thr172-AMPK and p-Ser239 VASP were utilized. GAPDH acts as a loading control. **(B)** Quantifications of the Western blots, related to **(A)**. *n* = 3, Student’s *t*-test, NS (not significant). **(C)** Representative Western blot of ctrl siRNA and AMPK siRNA-treated MCF10A cells. Specific antibodies against AMPK and GAPDH were utilized. Quantification of the siRNA experiments, *n* = 3, *p < 0.05, Student’s *t*-test. **(D)** AMPK interference significantly affects MLC-2 phosphorylation. Western blot analysis of cellular lysates from MCF10A cells, left untreated or treated with either leptin or adiponectin and siRNA against AMPK. Specific antibodies against p-Ser239-VASP, pp-Thr18/Ser19-MLC-2, total VASP or AMPK were utilized. GAPDH acts as a loading control. **(E)** Quantifications of the Western blots, related to **(D)** pp-MLC-2 and **(F)** pVASP; mean values ± SEM, *n* = 3; **p < 0.01, Student’s *t*-test. **(G)** median values of cell speed per trajectory of leptin 100 ng/ml with or without AMPK siRNA, results are from at least 62 cells per condition. *n* = 2, *p < 0.05, ***p < 0.001, One-way ANOVA, Bonferroni’s post-test. **(H)** the cell-to-cell bond length calculated similarly to Figure 2B, but in cells treated with leptin 100 ng/ml with or without AMPK siRNA, *p < 0.05, **p < 0.01, ***p < 0.001, One-way ANOVA, Tukey’s post-test.

Leptin has been associated with pro-mitogenic functions and induction of genes related to cancer invasion and metastasis (Yan et al., 2012; Kato et al., 2015; Figures 2C,D and Supplementary Figures S1A,B). To understand whether leptin could play a role in cell migration through actomyosin-mediated cellular forces, we studied its effect on the MLC-2 phosphorylation. For this, MCF10A cells were treated as in the previous assays (Figures 1, 2) and immunofluorescence stainings were performed to detect the levels of phospho-Thr18/Ser19 MLC-2 (Supplementary Figure S2B; here after referred as pp-MLC-2). Similarly, cellular lysates from such samples were utilized in Western blotting with specific antibodies against pp-MLC-2 (Figures 3A,B). These experiments showed that MLC-2 phosphorylation was induced by both adipokine treatments in a concentration dependent manner. Interestingly, adiponectin also showed moderate increase in the levels of pp-MLC-2, although it did not significantly impact morphology of actin-based structures (Figures 2A, 3A and Supplementary Figure S2B lower panel).

As MLC-2 phosphorylation and subsequent actomyosin contractility can be regulated by the activity of AMPK kinase (Miranda et al., 2010; Tojkander et al., 2015, 2018) and adipokines have been linked to the activation AMPK pathway (Libby et al., 2014; Kato et al., 2015), we wanted to assess the possible connection in between pp-MLC-2, AMPK activity, and adipokine treatments. The levels of P-Thr172-AMPK, indicating active AMPK, and its downstream target P-Ser239-VASP, were analyzed as above (Figures 3A,B). As with MLC, AMPK showed a concentration-dependent elevation in its Thr172-phosphorylation upon adipokine treatments, while P-Ser239-VASP showed some elevation in some of the experimental repeats, the average levels did not significantly differ from the ctrl P-Ser239-VASP levels (Figures 3A,B). To verify whether AMPK was indeed responsible for the elevated pp-MLC-2 upon adipokine treatments, we used specific siRNA against AMPK for depletion of this protein (Figure 3C). AMPK siRNA treatment had an effect on the cell shape and structure of the cytoskeleton on both single cell and monolayer conditions (Supplementary Figure S3). The cells were elongated and in addition, there were observed untypical “spiky” protrusions from the cell membrane in both monolayer and single cells. Further, the siRNA treatment of breast epithelial cells mitigated the leptin-induced effect on MLC-2 phosphorylation (Figures 3D,E), although the downstream target of AMPK, VASP, showed only a slight decreasing trend on phosphorylation of its Ser239 residue (Figures 3D,E). Adiponectin treatments did not show significant difference in between the groups (Figures 3D,E). Further, we applied AMPK gene interference (siRNA) in the motility assay and it abrogated the leptin-induced increased cell motility (Figure 3G). However, it has to be noted that the AMPK siRNA alone led to statistically significant decrease in the cell motility compared to the controls (Figure 3G). Additionally, to analyze the morphology of the cells upon these treatments, we performed cell-to-cell bond length analysis in the presence of AMPK siRNA and leptin (Figure 3H). However, AMPK siRNA-treatment alone significantly affected the morphology of the cells and the combined effect of leptin + AMPK siRNA is difficult to assess with this type of assay.

**FIGURE 4:**
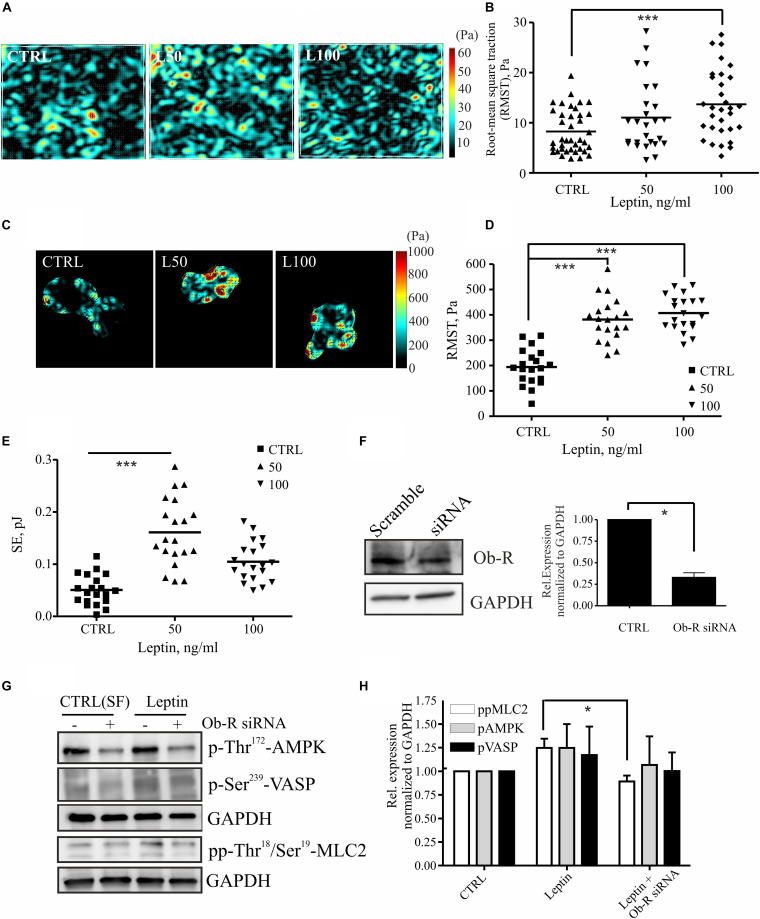
Leptin increases cell-substrate forces potentially through MLC-2 phosphorylation. **(A,B)** monolayer force measurements on silicone gels with 1 kPa stiffness after 72 h leptin treatment of monolayer MCF 10A cells. For **(B)** all values and the median are presented within each group, *n* = 26–39, ***p < 0.001, One-way ANOVA, Tukey’s post-test. **(C,D)** Traction forces exerted by cell doublets. Representative maps are shown in **(C)** and calculations of cell-exerted forces RMST in **(D)** and strain energy (SE) to the substrate in **(E)**. For **(D,E)** all values and the median are presented within each group, *n* = 19–20, ***p < 0.001, One-way ANOVA, Tukey post-test. **(F)** Efficiency of OB-R siRNA, mean values ± SEM, *n* = 4; Student’s *t*-test, *p < 0.05. **(G)** Cellular lysates of MCF10A cells, –/+ Ob-R siRNA and leptin (100 ng/ml) were utilized in Western blot experiments. Specific antibodies against pp-Thr18/Ser19 MLC-2, Thr172 AMPK and p-Ser239 VASP were used. GAPDH acts as a loading control. **(H)** Quantifications of the Western blot for pAMPK, pVASP, and pp-MLC-2, related to **(G)**; mean values ± SEM, *n* = 3; *p < 0.05, Student’s *t*-test, N.S.

MLC-2 phosphorylation regulates actomyosin contractility and concomitant cell-exerted forces that may be linked to higher invasion potential (Somlyo and Somlyo, 2000; Bershadsky et al., 2003; Huang et al., 2004; Mierke et al., 2008, 2011; Tojkander et al., 2011; Kraning-Rush et al., 2012; Raab et al., 2012; Aung et al., 2014). To study whether leptin treatment could also regulate actomyosin forces, we treated breast epithelial cell cultures as above and performed traction force microscopy (TFM) experiments. Increased motility of the leptin-treated cells was accompanied by a rise in the cell-substrate forces (Figure 4). Force measurements in multicellular setups, performed either with monolayers or doublets of breast epithelial cells, showed statistically significant increase in cell-substrate traction forces at higher concentration of leptin (Figures 4A–E). Based on this analyses, cell-exerted forces were increased after leptin treatment when compared to the serum-free controls, as is indicated by higher Root-Mean-Square Traction (RMST) and Straining Energy (SE). Taken together, treatment of mammary epithelial cells with leptin triggers MLC-2 phosphorylation at least partially in an AMPK-dependent manner. This potentially leads to higher actomyosin contractility and cell-exerted forces that may play a role in cell motility.

### Interference of Leptin Receptor Ob-R or Ca^2+^-Influx Abrogates Leptin-Induced MLC-2 Phosphorylation

Leptin mediates its effects through leptin receptor, Ob-R (Jardé et al., 2011; VanSaun, 2013). To confirm that leptin-induced effects on MLC-2 phosphorylation and consequent changes in actomyosin forces were compromised by the loss of Ob-R, we utilized specific siRNAs to deplete this cell surface receptor (Figure 4F). Interestingly, leptin-induced raise in pp-MLC-2 was lost upon Ob-R depletion (Figures 4G,H). However, we observed only a minor decrease in the phosphorylated forms of AMPK and VASP in ctrl versus leptin-treated samples (Figures 4G,H), although AMPK depletion seems to play a role in actomyosin dynamics in response to leptin treatment (Figure 3). It should also be noticed that Ob-R siRNA-treatment alone from control cells, decreased the levels of active AMPK, as shown in the Figure 4G upper lanes, confirming the previous findings that this receptor acts upstream of AMPK pathway.

**FIGURE 5:**
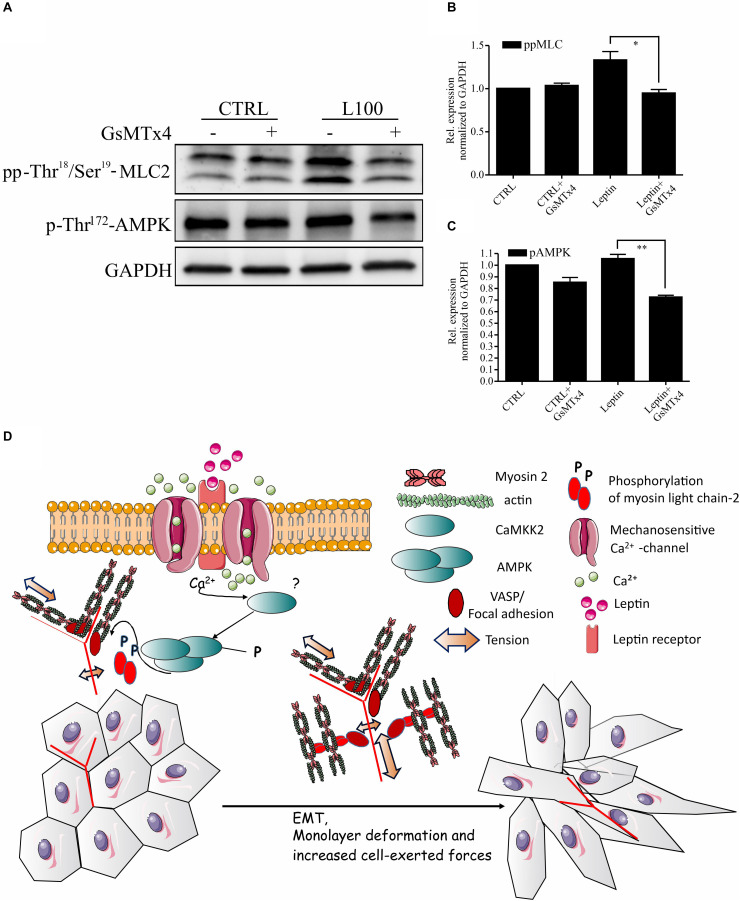
Inhibition of Mechanosensitive Ca^2+^ channels abrogates the effects of leptin on AMPK pathway. **(A)** Cellular lysates form MCF10A cells, left untreated or treated with leptin –/+ Ca^2+^ channel inhibitor, GsMTx4, were used for Western blotting. Specific antibodies against pp-Thr18/Ser19-MLC-2 and p-Thr172-AMPK were used. GAPDH acts as a loading control. **(B,C)** Quantifications of the pAMPK and ppMLC-2 levels, related to **(A)**. Results are mean values ± SEM, *n* = 3; *p < 0.05, Student’s *t*-test. **(D)** Hypothetical model: Leptin-induced changes in mammary epithelial cell cultures. Leptin is binding to its receptor (Ob-R), but also affects the function of some mechanosensitive Ca^2+^-channels. Through the co-operation of these plasma membrane-embedded proteins, leptin triggers intracellular signaling cascades, subsequently leading to increased actomyosin-mediated forces, EMT-like changes in cell morphology and enhanced migratory features.

As leptin is known to potentiate and co-operate with some ion channels (Pye et al., 2015; Domínguez-Mancera et al., 2017; Wu et al., 2017; Qiu et al., 2018) and AMPK is known to be downstream of some Ca^2+^-dependent kinases (Hong et al., 2005; Hurley et al., 2005; Rajakylä et al., 2020), we wanted to further assess the possible link in between leptin signaling and Ca^2+^ channels in actomyosin dynamics. To inhibit the function of several Ca^2+^ ion channels simultaneously, we utilized GsMTx4, a known inhibitor of a wide range of mechanosensitive Ca^2+^ influx channels (Li et al., 2015). Treatment of cells with this compound significantly diminished both the leptin-induced AMPK and MLC-2 phosphorylation (Figures 5A–C). These data suggest that the regulation of AMPK pathway and myosin 2-mediated contraction by leptin is taking place through a co-operative action in between leptin signaling and some yet unidentified calcium channel, as shown on the model figure (Figure 5D).

## Discussion

Obesity and adipose tissue-secreted adipokines are one of the risk factors for the development of metastatic breast cancer. Metastasizing cancer cells often have high contractility, linked to invasive migration (Mierke et al., 2008, 2011; Hanahan and Weinberg, 2011; Aung et al., 2014). Our key finding is that adipokine leptin is linked to the cellular force production through regulation of myosin light chain-2 phosphorylation and could thus in this way favor the invasive progression. Our results are summarized in the hypothetical model (Figure 5D).

Leptin and adiponectin have been shown to have contrary functions on breast cancer progression, leptin displaying tumor-promoting and adiponectin tumor-suppressive roles, f.i. through cell proliferation (Vona-Davis and Rose, 2007; Yan et al., 2012; Kato et al., 2015). Adipocytes have also been suggested to increase motility of breast cancer cells (Dirat et al., 2011; Jardé et al., 2011; Carter and Church, 2012). In our studies we aimed in understanding how these distinct adipocyte-secreted factors could play a role in invasive migration and observed that leptin-treated breast epithelial cells possessed higher migration speed in comparison to ctrl and adiponectin-treated cells (Figure 1). Additionally, leptin-treatment was associated with abnormal, elongated cellular morphology, accompanied with increased levels of EMT-associated proteins, including β-catenin, slug and snail (Figure 2 and Supplementary Figure S1). Snail is acting as a transcription factor during epithelial-to-mesenchymal transition and its increase together with the other upregulated markers in this context is suggestive for ongoing EMT (Medici et al., 2008; Phillips and Kuperwasser, 2014; Brabletz et al., 2018). EMT is one of the main pathways used by the transformed cancer cells during invasion (Hanahan and Weinberg, 2011; Brabletz et al., 2018). During that process, β–catenin is often upregulated through slug and snail (Medici et al., 2008; Phillips and Kuperwasser, 2014). Elevated β-catenin is connected with the internalization of E-cadherin, which weakens the cell-cell contacts and helps to compromise the structure of the epithelial monolayer (Howard et al., 2011). While we observed occasional cytoplasmic E-cadherin aggregates (Supplementary Figure S2A), the total levels of E-cadherin did not significantly alter and we could not detect a total disruption of the monolayers at least within the utilized time frame (Figure 2). It is possible that leptin-treated cells undergo only partial EMT. Olea-Flores et al. (2018) have reported such findings; they show leptin-triggered partial EMT within 24 h after treatment. Alternatively, these cells could utilize a collective migration mode (Trepat et al., 2009; Tambe et al., 2011; Serra-Picamal et al., 2015).

Further, we wanted to elucidate the mechanisms that leptin uses to potentiate motility of the breast epithelial cells. We detected that leptin triggers MLC-2 phosphorylation, indicating its role in actomyosin dynamics. As actomyosin forces play a role in cell migration and invasive migration in 3D (Raab et al., 2012), leptin could therefore affect invasive progression through its impact on the cellular force production. This was also supported by the traction force experiments that showed higher actomyosin forces in both cell doublet and monolayer setups (Figures 4A–E). Previously, the formation of contractile actomyosin bundles in migrating cells has been linked to AMPK pathway (Miranda et al., 2010; Tojkander et al., 2015, 2018) and AMPK pathway has been tracked downstream of leptin (Kato et al., 2015). Leptin could thus control cell actomyosin dynamics through AMPK. Interestingly, leptin-dependent induction in pp-MLC-2 was compromised by the depletion of AMPK (Figure 3). The effect of leptin on cell migration speed was also reversed by specific interference of the AMPK gene (Figure 3G), although AMPK siRNA alone had already significant impact on the migratory mode. Furthermore, we applied RNAi against Ob-R and tested its effect on AMPK and pp-MLC-2 (Figures 4F–H). Surprisingly, only leptin-induced pp-MLC was affected significantly by the depletion of Ob-R, while AMPK activity was reduced only to minor extent when comparing ctrl and leptin-treated cells upon Ob-R depletion. The results are suggestive of potentially parallel pathways, downstream of leptin that control actomyosin dynamics.

Adipocytes have been shown to display paracrine functions and adipokines can trigger other cellular receptors. EGFR, f.i. can be activated by leptin (Gilmore et al., 2002) and leptin may also impact the interplay in between IGF, insulin-like growth factor, and EGF (Vona-Davis and Rose, 2007). Leptin signaling can also co-operate with some cell surface Ca^2+^ channels (Gomart et al., 2013; Pye et al., 2015; Domínguez-Mancera et al., 2017; Wu et al., 2017; Qiu et al., 2018). Interestingly, AMPK has been shown to be activated in a Ca^2+^-dependent manner (Hong et al., 2005; Hurley et al., 2005; Rajakylä et al., 2020) and could be triggered by co-operative activation of some Ca^2+^ ion channels, downstream of leptin signaling. Leptin could thus impact pp-MLC-2 levels and actomyosin forces partially through Ca^2+^-dependent AMPK pathway. This was also supported by the finding that GsMTx4, an inhibitor of several Ca^2+^-channels, constrained the leptin-triggered increase in pp-MLC-2 (Figure 5). Previously, leptin has been shown to affect contractile features of rat aorta through TRPC6 calcium channels (Gomart et al., 2013). Ca^2+^ signaling has also been linked to the fat tissue-produced adipocytokine resistin and the increased invasiveness of MDA-MB-231 cells via higher levels of ezrin, a linker in between plasma membrane proteins and actin cytoskeleton (Lee et al., 2016).

Many cell surface Ca^2+^ channels have been reported to affect cell migration through cytoskeletal structures and be involved in the invasion of cancer cells (Lee et al., 1999; Bolanz et al., 2008; Dowd et al., 2017). Considering the interplay in between these channels and leptin signaling, leptin may have many potential ways to promote invasive progression through actomyosin structures. Which Ca^2+^-dependent channels are involved and how they are triggered by leptin signaling, needs to be further studied in the future.

## Materials and Methods

### Cell Culture

MCF10A cells were purchased from ATCC (ATCC^®^ CRL-10317^TM^) and maintained in DMEM/F12 supplemented as described by Brugge lab^1^ and elsewhere. The cells were subcultured before reaching 80% confluence. For adipokine treatments the MCF10A cells were serum-starved overnight (Yan et al., 2012). Leptin (#L4146) and adiponectin (#SRP4901) were purchased from Sigma-Aldrich and added to final concentrations of 50–100 ng/ml (leptin) and 25–250 ng/ml (adiponectin). The cells were incubated with adipokines for duration 24–72 h. As mechanosensitive Ca^2+^ channel inhibitor was used GSMTx4 (#ab141871, Abcam) in concentration 2.5 μM for 16 h.

For *siRNA silencing*, 25 nM ON-TARGET plus SMARTpool^TM^ siRNA library AMPK(#J-005027-06 Dharmacon, GE Healthcare) or Ob-R (leptin receptor, #L-008015-00-0005 Dharmacon, GE Healthcare) were transfected into cells on 35 mm plates by using RiboJuice transfection reagent (#71115, Novagen) according to the manufacturer’s instructions. Cells were incubated for 72–96 h for efficient depletion of the target protein.

### 3D Spheroid Cultures

They were created as described by the Brugge lab (see footnote) with modifications. We used 35 μl Matrigel^TM^ Growth Factor Reduced (GFR) Basement Membrane Matrix (#354230, Corning) to pre-coat the slides, 5,000 cells per well. The cultures were fed with 4% Matrigel in complete DMEM/F12 every 5 days and cultured for totally 14 days.

### Western Blotting

MCF10A cells were lysed with Lysis Buffer containing 50 mM Tris, HCl pH 8.0, 150 mM NaCl, 1% Triton X-100 with Protease inhibitor Cocktail Set III (#539134, Calbiochem), Phosphatase Inhibitor Cocktail Set II (#524625, Calbiochem) Millipore, Merck Life Science OY, Espoo, FI). The protein concentration was measured according the Bradford method (#5000205, BioRad). The protein solution was diluted in 4x SDS loading buffer (40% glycerol, 4% SDS, 250 mMTris-HCl, 3% DTT, bromphenol blue) and 10 or 30 μg per line loaded on 4–15% Mini-PROTEAN^®^ TGX^TM^ Precast Gels (#456-1084, BioRad). Used protein ladder was from BioLabs (#P77125). The separated proteins were transferred on Immobilon-P (#IPVH00010, Merck) PVDF membrane using semi-dry (#170-3940, Bio-Rad) or wet transfer systems. The non-specific binding was blocked with blocking buffer (5% skimmed milk (or BSA), 0.1% Tween-20 in PBS) for 1 h at room temperature. The membranes were incubated with primary antibodies in dilutions: α-rabbit Phospho-AMPK (Thr172) (#2335, CST) and α-rabbit Phospho-MLC-2 (Thr18/Ser19) (#3674, CST), α-rabbit Phospho-VASP (Ser239) (#05-611, Millipore), α-rabbit E-cadherin (#3195, CST), α-rabbit slug (#9585, CST), α-rabbit AMPK (#SAB4502329, Sigma-Aldrich), α-rabbit Beta-catenin (#8480, CST), α-rabbit Ob-R (#ab5593, Abcam) and α-rabbit GAPDH (G9545, Sigma) overnight at 4°C. The membranes were washed with 0.1% PBS-T and incubated with secondary HRP- conjugated antibody (Anti-Mouse/Anti Rabbit IgG, CST) for 1 h at room temperature. After washing with 0.1% PBS-T membranes were incubated with Luminata Crescendo Western HRP substrate (#WBLURO100, Millipore) and visualized on LAS-3000 Imaging System (Fujifilm Corporation, Tokyo, Japan).

### Immunofluorescence

The MCF10A cells were plated on laminin-pre-coated coverslips as single cells or in concentration to form monolayers. After treatment and incubation, the cells were fixed with 4% paraformaldehyde for 20 min at RT then washed three times with PBS. Permeabilization and blocking of non-specific binding was performed by 5% FCS (GIBCO) and 0.3% Triton X-100 (Sigma-Aldrich) in PBS at RT for 30 min. Primary antibodies were added to the cells diluted in 1% FCS in 0.3% Triton-X100-PBS: rabbit anti-E-cadherin 1:75 (Cell Signaling Technologies) for 1 h at RT as 25 μl per coverslip which was inverted on Parafilm (Bemis Company) in moist chamber. The samples were incubated with Phalloidin-647 (#A22287) 1:200 for 30 min at RT. Wash 3x PBS. Secondary anti-mouse Alexa-568 (#A11031) or anti-rabbit Alexa-488 (#A11034) conjugated antibody 1:300 (Molecular Probes) for 30 min at room temperature. DAPI nuclear stain was applied in 1:2,000 (from 0.1 μg/ml stock) for 10 min at RT. The coverslips were dipped in Milli-Q water and mounted with Mowiol-DABCO (Millipore and Sigma-Aldrich) mounting medium 8–10 μl/slide, max. 4 coverslips per slide.

### Immunofluorescence for 3D

The spheroids were fixed with 2% PFA for 20 min at room temperature, followed by 10 min permeabilization with 0.25% Triton-X100 in PBS. Unspecific binding sites were blocked with 10% goat serum in IF buffer (0.1 % BSA, 0.2% Triton-X100, 0.05% Tween 20, 7.7 mM NaN_3_ in PBS) for 2 h at room temperature. The primary antibodies were diluted in blocking buffer 1:100 and incubated over night at 4°C. The slides were washed three times 10 min with gentle rocking and incubated with secondary antibodies (Goat Anti-mouse Alexa 568, Goat Anti-Rabbit Alexa 488, Alexa Fluor Phalloidin 647 in 1:300 dilution for 45 min at room temperature in the dark. The nuclei were counterstained with DAPI (0.05 ng/ml) for 10 min at room temperature. After wash with PBS, the plastic chambers and silicone gasket were removed. Finally, the slides were briefly rinsed with distilled water and mounted with Mowiol mounting medium. 2D cultures were imaged with LEICA DM6000B using 20x/0.7 HC PL APO CS wd = 0.59, 40x/1.25 − 0.75 HCX PL APO CS Oil wd = 0.10 and 63x/1.40 − 0.60 HCX PL APO Lbd.bl. Oil wd = 0.10 objectives.

### Traction Force Microscopy

MCF10A cell doublets were cultured for 3 h on collagen-1-coated polyacrylamide (PAA) gel substrates (elastic modulus = 1 or 4 kPa) that were coated with sulfate fluorescent microspheres (diameter = 200 nm, Life Technologies). Using an inverted fluorescence microscope 3I Marianas imaging system, containing a heated sample chamber (+37°C), controlled CO_2_, and 63 × /1.2 W C-Apochromat Corr WD = 0.28 M27 objective (3I intelligent Imaging Innovations, Germany) (Marinković et al., 2012), images of cells and of the fluorescent microspheres directly underneath the cells were imaged during the experiment and after cell detachment with trypsin. By comparing the fluorescent microsphere images before and after cell detachment, we computed spatial maps of cell-exerted displacement. With knowledge of the displacement field and that of the substrate stiffness, we computed the traction field using the well-established method of constrained Fourier transform traction microscopy (Butler et al., 2002; Krishnan et al., 2009). Similarly, monolayer traction force microscopy was performed on NuSil^®^ (#8100, NuSil Silicone Technologies) gels crosslinked with Sylgard 184 agent (#1673921, Dow Corning) (gel elastic modulus = 1 kPa) embedded with fluorescent microspheres. Detailed protocol is described in Yoshie et al. (2017).

### Random Motility Assay

2 × 10^4^ MCF10A cells were plated in 24-well plates and treated with adipokines with or without AMPK siRNA as described earlier. On the next day the plate was placed in CellIQ imaging device (CM technologies, Tampere, Finland) and images were taken every 30 min for 24 h. The cell trajectories were followed with CellIQ Analyser (CM technologies) and the parameters of the cell motility as speed per trajectory were calculated using the manual scoring option of the software.

### Monolayer Analyses

Tissue Analyzer plugin (Tissue Analyzer v1.0, Copyright 2007–2015 by Aigouy et al., 2016) for Fiji-ImageJ 1.52p (National Institutes of Health, Bethesda, MD, United States^2^, 1997–2019) software was used to segment the monolayers to cells using the watershed algorithm. Then we measured the cell area and the cell-to-cell bond lengths. Briefly, we cropped same size areas from the monolayers and performed bond recognition using the Tissue Analyzer built-in function. Cell bonds are pixels shared by exactly two cells and their length is presented in μm. Bounds are defined by the two cells that share them and the increase in their length is a measure for cell elongation. Each image was inspected and small false-recognized bonds were removed manually. The analysis was finalized and cell bond and cell size (data not shown) parameters were scored in at least 100 cells per treatment.

### Statistical Analysis

The results were represented as mean values ±SEM for all the methods except the random motility assay and the spheroid volume where there was used the median value ±SEM. The ANOVA tests were performed with the statistical package included in Prism 4 (GraphPad Software Inc., United States).

## Data Availability Statement

The original contributions presented in the study are included in the article/Supplementary Material, further inquiries can be directed to the corresponding author/s.

## Author Contributions

AA has performed most of the experiments in Figures 1–5 and Supplementary Figures S1–S3 and participated in writing the manuscript and preparing the figures. TK has performed Western blotting experiments for Figures 3, 4. NS and RK have participated in the traction force experiments. AA and ST designed the experiments, wrote the manuscript, and prepared the figures. All authors contributed to the article and approved the submitted version.

## Conflict of Interest

The authors declare that the research was conducted in the absence of any commercial or financial relationships that could be construed as a potential conflict of interest.

## Footnotes

1 https://brugge.med.harvard.edu/protocols

2 http://imagej.nih.gov/ij/
