# Supplementary material for: Adipokine Leptin Co-operates With Mechanosensitive Ca2 +-Channels and Triggers Actomyosin-Mediated Motility of Breast Epithelial Cells
Source: Front Cell Dev Biol. 2021 Jan 6;8:607038. doi: 10.3389/fcell.2020.607038 (PMC7815691; doi:10.3389/fcell.2020.607038)
Supplement: Supplementary file 1 [file Data_Sheet_1.pdf]

SUPPLEMENTARY MATERIAL

SUPPLEMENTARY FIGURES AND SUPPLEMENTARY FIGURE LEGENDS

Supplementary Figure 1

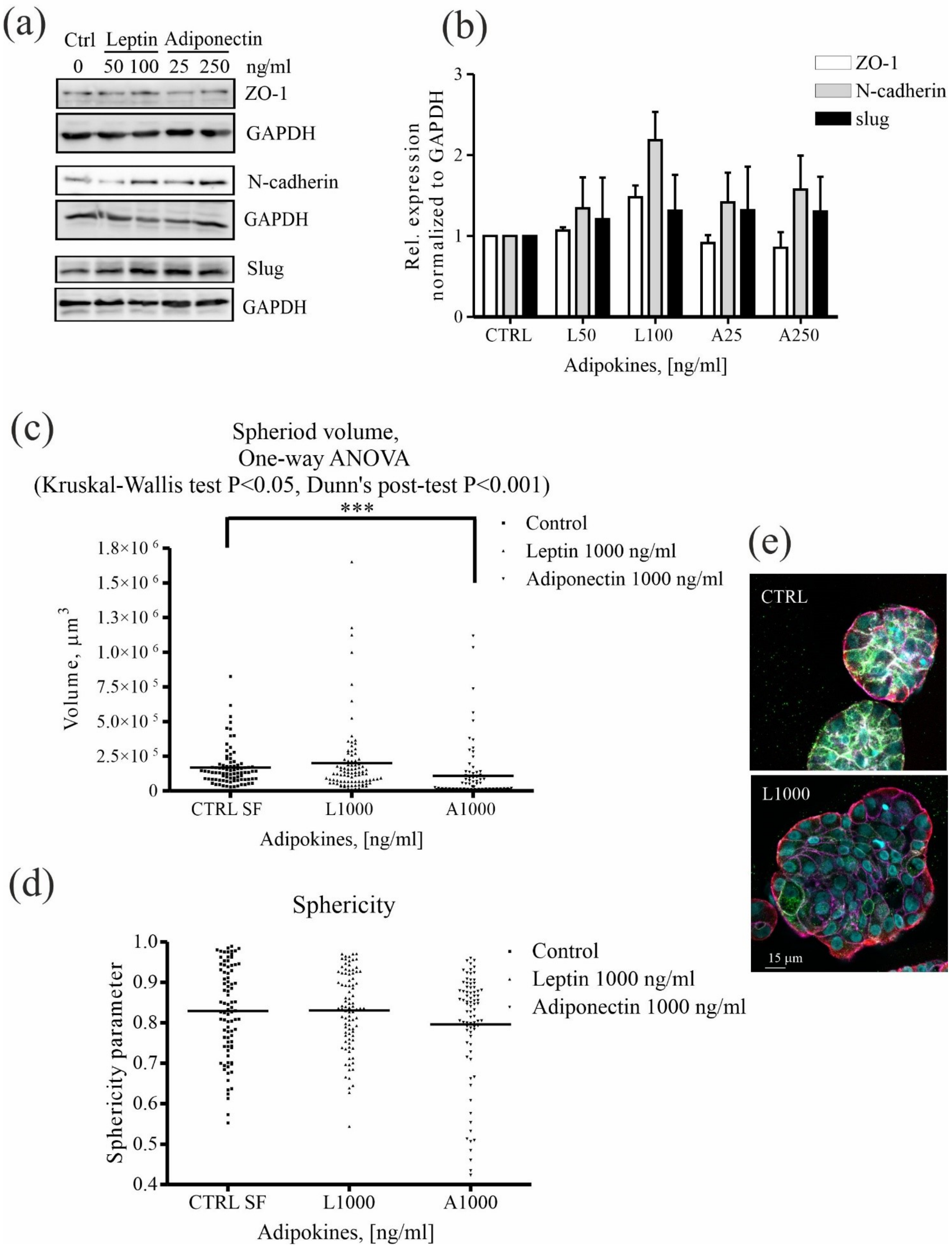

Supplementary Figure 1. Leptin induces EMT and abnormal 3D morphology. (a) Cellular lysates from ctrl, leptin-or adiponectin-treated MCF10A cells were utilized in Western blotting with specific antibodies against ZO-1, N-cadherin and slug. GAPDH acts as a loading control. (b) Quantification of the Western blots, related to (a). n=3 Student's t-test, N.S. for all. (c) Spheroid volume and (d) sphericity were assessed from 3D matrigel cultures from ctrl, leptin-or-adiponectin-treated spheroids that were fixed and stained as in Fig. 2e. Measurement of spheroids was performed with Imaris 9.5 software (Oxford Instruments, UK). (e) Additional example of mammosphere structure variation after 1000 ng/ml leptin. Scale bar 15 μm.

Supplementary Figure 2

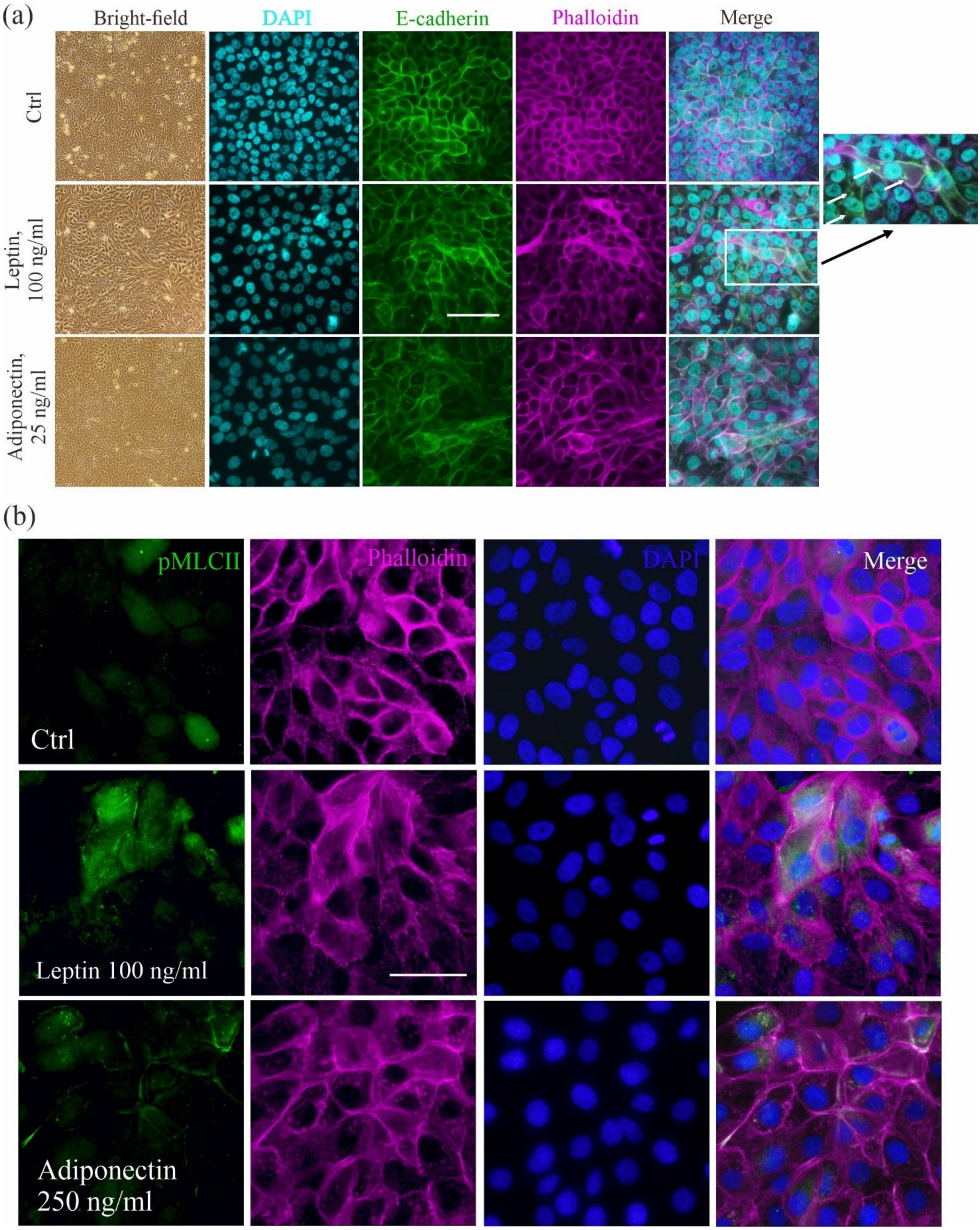

Supplementary Figure 2. Leptin and adiponectin effects on cell morphology (a) and MLC-2 phosphorylation (b). (a) Cell morphology and E-cadherin expression in MCF10A cells treated with 100 ng/ml leptin and 25 ng/ml adiponectin. White arrows point internalized E-cadherin aggregates Scale bar 50 µm. (b) Immunofluorescence stainings of leptin- (middle panels) and adiponectin- (lower panels) treated cells (72 hours after treatment). Specific antibody against pp-Thr18/Ser19-MLC-2 was used. Phalloidin was used to detect actin cytoskeleton and DAPI nuclei. Scale bar 50 µm.

Supplementary Figure 3

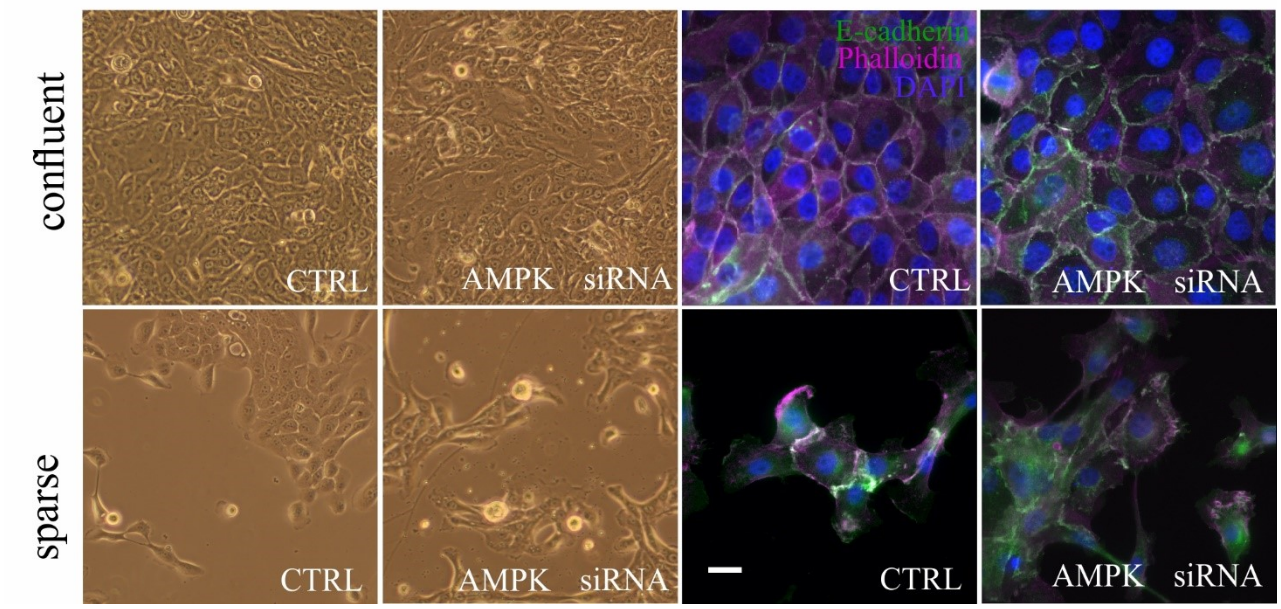

Supplementary Figure 3. Impact of AMPK siRNA on the breast epithelial cultures. Immunofluorescence stainings of confluent and sparse ctrl and AMPK siRNA treated MCF10A cells. Specific antibody against E-cadherin was used, phalloidin was used to detect actin cytoskeleton and DAPI nuclei. Scale bar 20 µm.
